# The microbiome mutiny hypothesis: can our microbiome turn against us when we are old or seriously ill?

**DOI:** 10.1186/s13062-014-0034-5

**Published:** 2015-01-14

**Authors:** Lajos Rózsa, Péter Apari, Viktor Müller

**Affiliations:** MTA-ELTE-MTM Ecology Research Group, Eötvös Loránd University and the Hungarian Academy of Sciences, Pázmány s. 1/C, 1117 Budapest, Hungary; Department of Evolutionary Zoology and Human Biology, University of Debrecen, Debrecen, Hungary; Department of Plant Taxonomy, Ecology and Theoretical Biology, Eötvös Loránd University, Pázmány s. 1/C, 1117 Budapest, Hungary; MTA-ELTE Theoretical Biology and Evolutionary Ecology Research Group, Eötvös Loránd University and the Hungarian Academy of Sciences, Pázmány s. 1/C, 1117 Budapest, Hungary; Parmenides Center for the Conceptual Foundations of Science, Pullach, Munich Germany

**Keywords:** Evolutionary medicine, Facultative virulence, Microbiome, Host-pathogen communication, Pathogenesis

## Abstract

**Background:**

The symbiotic organisms of the healthy microbiome tend to be harmless or even beneficial for the host; however, some symbionts are able to adjust their virulence in response to external stimuli. Evolutionary theory suggests that optimal virulence might increase if the mortality of the host (from unrelated causes) increases.

**Presentation of the hypothesis:**

We hypothesize that microorganisms of the human microbiome may be capable of a coordinated phenotypic switch to higher virulence (“microbiome mutiny”) in old or seriously ill people, to optimize their transmission under the conditions of increased background mortality. This proposed virulence shift might contribute to the death of old or seriously ill people even in the absence of apparent disease.

**Testing the hypothesis:**

Testable predictions of the hypothesis include increased expression of virulence factors in isolates of the same species of the microbiome obtained from ailing/old versus healthy/young individuals, and the existence of microbial mechanisms to assess the general condition (background mortality) of the host. Such tests are going to be important to distinguish the cases of “microbiome mutiny” from the situation where opportunistic infections or increased effective virulence arise from relaxed immune control in ailing or old individuals in the absence of changes in the symbionts/pathogens.

**Implications of the hypothesis:**

Elucidating this potential mechanism might open up new possibilities for the clinical management of age related health issues and critical injuries or disease. Targeted prophylaxis against the microbes capable of virulence shifts could break the harmful feedback loop between deteriorating health and the “mutiny” of the microbiome.

**Reviewers:**

This article was reviewed by Eugene V Koonin, Neil Greenspan and Michael Gilchrist.

## Background

From an evolutionary point of view, virulence is defined as the reduction in the lifetime reproductive success of host individuals owing to infection with a symbiont/parasite. A full continuum exists from lethal pathogens to obligate mutualists, and the lines between parasitism and mutualism are often blurred [1,2]; some species are even able to adjust their virulence reacting to a changing environment [3,4]. Differences in the speed and extent of the symbionts’ reproduction can directly contribute to the variability of their virulence. Slower growth and multiplication of symbionts is associated with a lighter metabolic burden and smaller costs of immunity and collateral damage. However, as expressed in the ‘trade-off hypothesis of virulence’ [5], slower replication is also likely to result in reduced transmissibility of the symbiont over a unit time, due to lower densities in the infected individual. General theoretical considerations suggest that symbionts evolve towards an optimal virulence that maximizes their transmission over the entire life cycle of infection [6,7]. Optimal virulence depends on the life history traits of both the host and the symbiont species, and both highly lethal, and benign or mutualistic (“negative virulence”) interactions can be evolutionarily stable strategies. The symbionts adapted to long-term persistence in their hosts are characterized by low (or negative) virulence to allow the hosts to survive through longer periods [8,9].

Virulence is not only subjected to quick evolutionary changes, but some pathogens are also capable of exhibiting phenotypic plasticity in virulence traits [3]. For example, some parasites exhibit low virulence when facing “cooperative” hosts (that mount weak defense) and high virulence in “non-cooperative” hosts (that mount vigorous defense) [10].

The human microbiome is a taxonomically diverse mass of bacteria (and to a lesser extent, also archaea and fungi) living in and on both healthy and diseased humans [11]. This microbial community is widespread across all human body surfaces, namely the skin, nasal and oral cavities, genitals, lungs, and is particularly abundant within the intestines. In healthy humans, most members of the microbiome exhibit zero or negative virulence thus comprising neutral or mutualistic relationships, respectively. However, this mode of benign coexistence likely evolved under the conditions of a healthy host—representing the majority of the lifespan of humans. Human mortality exhibits a pattern of low mortality rates during most of the lifespan, but steep increase at old age [12], and episodes of serious injury or illness can substantially raise mortality also at younger age. Evolutionary theory suggests that increasing mortality might increase the optimal virulence of a symbiont [13,14]: killing the goose that lays the golden eggs might not be such a bad idea if the goose is going to die soon, anyway.

## Presentation of the hypothesis

Extending earlier work [13,15], we propose that symbionts of the human microbiome might shift to higher virulence (or from mutualism/commensalism to parasitism/pathogenesis) as background mortality increases steeply at the end of human lifespan or due to serious injury or illness. As the option for long-term persistence becomes increasingly limited, the symbionts are likely to benefit from increasing host exploitation rates so as to maximize chances of immediate transmission. A recent survey of the human microbiome found that a large number (>50 species) of opportunistic pathogens are widely prevalent in the microbiota of healthy individuals [11], suggesting that many species of the microbiome (sometimes referred to as ‘pathobionts’ [16]) are indeed able to switch facultatively to higher virulence. Switches to higher virulence might also occur if mortality increases due to other reasons, e.g., severe injury or infection, provided the symbionts are able to detect such changes in the host’s condition (which is not unreasonable to assume: see below).

Observations consistent with our hypothesis include the increased incidence of diarrhea in old age [17]; the reactivation of herpesviruses in aging [18] or after helminthic co-infection [19]; and the increased risk of common infections (including those with opportunistic pathogens) in aging individuals [20] or in patients with type 1 and type 2 diabetes mellitus [21]. Of note, aging has the strongest impact on the incidence of pneumonia and urinary infections [20], which are often caused by opportunistic pathogens and which both provide a simple mechanism of increased shedding of the infectious microorganisms (coughing and bacteriuria) when the replication of the pathogens accelerates. Admittedly, many of these observations could also be explained by compromised immune control over the (potential/opportunistic) pathogens in aging or diseased individuals, rather than a change in the behavior of the symbionts/commensals.

We further argue that virulence shifts across the microbiome might occur in a synchronized fashion. Whenever certain members of the microbiome switch to higher levels of virulence, the expected lifespan of the host decreases further. Sensing either the further decline in the health of the host, or directly the increased replication or virulence of the other symbionts co-inhabiting the same person might trigger further microorganisms to switch to increased virulence. As a consequence, the switching of some major components of the microbiome to higher host exploitation rates likely provokes a chain reaction of virulence shift across the whole community, somewhat analogous to a ‘regime shift’ (sudden switch between alternative states) in ecosystems [22].

The proposed hypothesis of “microbiome mutiny” depends on the assumption that symbionts are able to obtain reliable information on the health and life expectancy of the host that they inhabit. This might occur, e.g., by sensing increasing oxidative stress [23] or other molecular or physiological markers of senescence [24]. Impaired health due to injury or disease might be sensed based on systemic markers of inflammation [25], while an increased level of heat shock proteins can indicate stress and reduced health in both aging and disease [26]. Remarkably, stress-induced host signaling molecules have been shown to induce a high-virulence phenotype in experimental *Pseudomonas aeruginosa* infection of mice [27]. In addition, symbionts might detect direct or indirect clues of the presence and activity of other (potentially) disease-causing organisms, sensing secreted diffusible molecules in the frame of a microbial community-wide quorum-sensing system [28,29], or host immune status manipulated by co-infections [19]; these and other mechanisms of sensing signals from both the host and other members of the microbiota are reviewed in [30]. The ability to adjust virulence in response to external stimuli does not seem to be particularly difficult to evolve: even a simple bacteriophage could quickly be adapted to conditional virulence under experimental conditions [31].

Finally, we note that the evolution of host-health dependent virulence may depend on some aspects of the life history of the hosts. For example, both healthy and ailing hosts must be sufficiently frequent to create alternating selection regimes that favor low and high virulence in the symbiont, respectively. If the symbiont spends very little time in ailing hosts, then this rare selection regime may be insufficient for the emergence and/or maintenance of adaptive virulence mechanisms. Furthermore, the population structure of the microbiome across host individuals may also be important: if host individuals live in close-knit groups that share the same microbiome, then a temporary increase in transmissibility from old or ailing individuals may bring little benefit for a symbiont that is already present in the other members of the group. However, while such a close-knit structure may have characterized hunter-gatherers, increasing population density and mixing in recent human history has created conditions where a huge inter-individual diversity of microbiomes [11] can now be readily exchanged. The composition of microbiomes is still more similar within than between families, but family membership accounts for only about 20% of the compositional variation in fecal samples of the gut microbiome [32]. We summarize the conditions required for the evolution of mechanisms of “microbiome mutiny” in Table 1.

**Table 1:** **A list of conditions required for the evolution of mechanisms of “microbiome mutiny”**

**Condition**	**Significance**	**Evidence**
Trade-off between rate of transmissibility (infectiousness) and host life span	Required to make “optimal virulence” dependent on the background rate of host mortality	Symptoms (morbidity) can aid transmission: diarrhea, coughs, bacteriuria
Detection of host health (mortality)	Required to sense changes in the selection regime (due to changing host mortality)	Bacterial sensing of host stress molecules turns on virulence phenotype [27]
Alternating selection regimes (mortality pattern) in the host population	Required to impose selection pressure from both regimes (favoring low and high virulence)	Extended aging in humans
Variability of microbiome composition among connected host individuals	Required to make between-host spread (strongly) dependent on instantaneous transmissibility	Large compositional variability in the human population [11], even within families [32]

In turn, humans (and other affected host species) might have evolved counter-adaptations to reduce the risk of “microbiome mutiny”, either by interfering with the microbial sensing of declining host health, or by creating conditions that favor symbionts that are not prone to virulence shifts but might suppress potentially pathogenic species [33]. Remarkably, systemic exposure to bacterial products triggers in mice the production of fucosylated proteins that can be metabolized by the gut microbiome and that have been shown to down-regulate virulence genes in gut bacteria [34].

Selection to oppose a microbiome mutiny in old age is likely to act in humans in particular, where living a long and healthy life even after the reproductive phase of life is adaptive because elder individuals provide valuable altruistic help to their relatives. In prehistoric ages, elderly people probably played an outstanding role in gathering, storing and distributing knowledge through the community. Moreover, grandparents can provide care specifically for their grandchildren—a social system unique to humankind [34].

## Testing the hypothesis

Our hypothesis predicts a secondary increase in host mortality due to “microbiome mutiny” when an independent cause (old age or illness) has generated a primary increase from the healthy baseline. Because we are typically unable to predict the primary increase quantitatively, it is not possible to assess the existence of a secondary increase solely on the basis of observed mortality rates. Even “per pathogen pathogenicity” (introduced as “per parasite pathogenicity” in [35]) is not a reliable marker, because the sensitivity (tolerance) of an immune compromised host to the same level of symbiont /pathogen load may also change independent of a virulence change in the symbiont/pathogen. The validation of the hypothesis will therefore have to rely on the discovery (and possible targeted manipulation) of the microbial mechanisms of “microbiome mutiny”. Direct tests of the hypothesis should include comparisons of the expression of virulence factors and in vitro measures of virulence between isolates of the same species of the microbiome obtained from healthy/young and ailing/old individuals. Our hypothesis predicts that increased virulence is not merely a consequence of increased replication due to relaxed immune control, but is also associated with the upregulation of virulence factors. We note, however, that the two mechanisms are not mutually exclusive and might, in fact, act in synergy: relaxed immune control might allow the replication of the microorganisms to higher levels, which might then turn on virulence mechanisms by quorum sensing [29,36]. Once putative mechanisms of microbiome mutiny have been identified, strong validation for the hypothesis can be derived from targeted inhibition of these mechanisms, which should then result in the maintenance of the low-virulence phenotype and reduced mortality, without directly interfering with the primary cause of increased background mortality or the growth of the targeted facultative pathogen. The feasibility of this approach has already been demonstrated in an animal model of *P. aeruginosa* infection displaying facultative virulence [37].

From the host side, our hypothesis likely applies to long-lived species that have a steep increase in mortality at the end of their life span. Large mammals and some birds fall in this category, while long-lived reptiles and plants tend to have flat or even decreasing mortality rates at higher ages [12]. From the symbionts’ side, the hypothesis applies to species that are able to establish long-term persistence in the host, and that have the capacity to increase their replication rate and transmissibility from the level attained in a healthy host. This might include normally harmless or beneficial members of the microbiome, with a probable dominant role of the gut microbiome that harbors the greatest microbial mass and taxonomic diversity, and that has an “easy route” to higher immediate transmissibility and virulence in the form of diarrhea. In addition, the hypothesis might also apply to persistent and/or latent infections with herpesviruses, hepatotropic viruses, spirochaetes (Lyme disease, syphilis), parasitic protozoa (malaria, toxoplasmosis), and even parasitic worms.

Finally, we note that increasing virulence in aged or chronically ill individuals might also occur by genetic changes (adaptive evolution) in infections with fast replicating organisms [38,39]. However, within-host evolution will play a role only if increased virulence confers a selective advantage to the symbiont/pathogen within the host, while our argument is based on changes in the degree of virulence that maximizes transmission, i.e. on selection at the between-host level. The two levels of selection may nonetheless be connected in some cases: in addition to conditional phenotypic plasticity, the capacity for microbial mutiny can also emerge by the evolution of “enabling constraints” that create a high-probability trajectory of evolution towards increasing virulence within the host when the appropriate conditions occur. The direct causes of the virulence shifts (phenotypic plasticity/facultative virulence vs. adaptive evolution/genetic changes) can be distinguished by screening for causative mutations.

## Implications of the hypothesis

Our hypothesis posits that at the moments when our health is most fragile due to old age or severe illness, our condition can be further exacerbated by the “treachery” of our previously benign microbiome. In terms of failing health, “whosoever hath not, from him shall be taken away even that he hath” (New Testament, Matthew 13:12). However, even if this phenomenon is widespread, the effect of shifting virulence might easily have gone unnoticed, because the declining health of the individuals could be attributed to the original condition that triggered the change in the microbiome.

Elucidating this potential mechanism might open up new possibilities for the clinical management of age related health issues and critical injuries or disease. Targeted prophylaxis against the microbes capable of virulence shifts could break the harmful feedback loop between deteriorating health and the “mutiny” of the microbiome. In this regard, the widespread emergency practice of applying preventive antibiotics even in the apparent absence of known pathogens, though criticized as a source of selection for antibiotic resistance, might not be such a bad idea [40]. A more detailed knowledge of the virulence shift mechanisms might in the future allow us to target directly the mechanisms responsible for the virulence switch.

If virulence shifts occur in a chain reaction of several species of the microbiome, then the altered regime of increased virulence might remain stable or might take a long time to revert even if the injury or disease that triggered the switch is cured. The negative effect of the symbionts already in “virulence mode” might suffice to keep each of them in this state, which might prolong convalescence. Of note, patients who survive to hospital discharge after sepsis remain at increased risk for death in the following months and years [41]. This state of ‘chronic critical illness’ can also be triggered by other acute episodes of illness (e.g., acute lung injury), and its maintenance seems related to persistent systemic inflammation [25], which might act by maintaining the microbiome in the altered regime of increased virulence. Targeted interventions might be able to break this state and quickly return the microbiome to the healthy regime of benign coexistence. The hypothesis also predicts that symbionts transmitted from old or seriously ill individuals might display higher initial virulence in the new host until virulence is “reset” to the baseline level optimal in healthy individuals.

One of the direct causes of mortality in cancer is wasting, and microbiome-induced inflammation has been implicated as a possible action mechanism [42]. This raises the possibility that “microbiome mutiny”, triggered by the primary condition of cancer, may contribute also to cancer mortality.

Virulence shifts might also play a role in the increased mortality observed after the loss of a lifelong partner, which occurs sometimes without specific pathological reasons [43]. Abruptly increasing levels of stress and associated psychosomatic changes [44] might trigger the shift to higher virulence in the microbiome, although other pathological processes, e.g., takotsubo cardiomyopathy (“broken heart syndrome”) [45], might also play an important role in the mechanism of this process. Remarkably, a recent study found that increased levels of norepinephrine (indicating emotional of physical stress) can induce the dispersion of *P. aeruginosa* biofilms [46], which might trigger acute cardiovascular disease: bacteria might even have to do with a broken heart.

Mortality might occur without a specific apparent cause also in the elderly: this is referred to as ‘debility not otherwise specified (NOS)’ or ‘failure to thrive (FTT)’ [47]; in colloquial Hungarian a specific expression exists, spelled as ‘végelgyengülés’ and loosely translated as “terminal frailty”, for this generic cause of death. Virulence shifts of the microbiome might contribute also to this gradual nonspecific weakening at the end of life.

Finally, the analysis of the microbiome from old but healthy individuals might help us identify microbial species that are less prone to virulence shifts, or host factors that can prevent the shifts. In contrast, the transmission of symbionts from individuals of failing health should be prevented, as these symbionts are more likely to have switched to higher virulence.

## Reviewers’ comments

We thank the reviewers for their helpful comments, suggestions and insights that have helped us improve our manuscript.

### Reviewer #1: Professor Eugene V. Koonin

This paper develops the “microbiome mutiny” hypothesis according to which the virulence of multiple opportunistic bacterial pathogens in the mammalian microbiome increases with a sharp increase of host mortality caused by aging and/or severe illness. The hypothesis is perfectly sensible readily testable and compatible with numerous observations.

Authors’ response: *We thank the reviewer for his appreciation of our hypothesis.*

Indeed it is common knowledge that old and sick people are subject to all kinds of opportunistic infections (pneumonia urinary infections and more). Certainly so are immuno-compromised individuals e.g. AIDS patients. Hence the alternative hypothesis that the susceptibility to infection in old age and illness is caused by the decline of immunity. The authors acknowledge this possibility but only in passing in the middle of the article. Hopefully this review makes this alternative more apparent. The two scenarios certainly are compatible and in my opinion both most likely contribute to the frailty of aging and ailing individuals.

Authors’ response: *We fully agree that this alternative (and parsimonious) explanation needs to be acknowledged, and have added this hypothesis, and the need of empirical tests to distinguish it from cases of “microbiome mutiny”, to the abstract to call attention to the alternative at the earliest opportunity. We have also added new text on the difficulties of distinguishing between the two alternatives to the Testing the hypothesis section.*

The best thing about this hypothesis is that it is likely to stimulate focused comparisons of the microbial populations and microbial transcriptomes from individuals of different ages (best of all longitudinal studies). All in all I find this a very interesting and useful article.

Authors’ response: *We share the hope that our hypothesis is going to stimulate empirical studies to validate the importance of the concept.*

### Reviewer #2: Professor Neil Greenspan

I found this Hypothesis article well-written interesting and reasonably persuasive. My enthusiasm for publication is high.

Comments to Authors:

Rozsa et al. propose an interesting thought-provoking and evolutionarily informed hypothesis pertaining to the relationship between host health as influenced by aging or other factors such as injury or infection and the magnitude of expression of virulence-related genes in bacteria normally present in host microbiomes. I found it both easy and pleasurable to read.

The authors clearly and logically present their argument that residents of the microbiome might detect chemical indicators of host health and appropriately modulate virulence-associated gene expression to optimize transmissibility provide limited preliminary supporting evidence from prior studies and offer experimentally testable propositions. Also to their credit the authors have considered explanations other than their preferred one for some of the previously published supportive observations that they cite.

I would not be surprised if this article stimulates useful new experimental investigations of host health-related variations in virulence gene expression among residents of the microbiomes in humans and experimental animals. In any case this article should be of interest to microbiologists infectious disease specialists gerontologists and a broad range of clinicians in a variety of disciplines given the broad impact of the microbiome on human health and disease pathogenesis.

Authors’ response: *We thank the reviewer for his very positive assessment of our hypothesis and supporting evidence.*

### Reviewer #3: Professor Michael Gilchrist

General Criticisms & Remarks

This is a readable paper that briefly explores an interesting and potentially very important aspect of the host-microbiome interaction. Most of my criticisms reflect suggestions for improving the paper rather than any fatal flaws.

Authors’ response: *We thank the reviewer for his appreciation of our hypothesis and his constructive criticism that has helped us refine the manuscript.*

I will, however, note that the authors are bit ‘adaptationist’ in their perspective. In order to effectively test this idea, we’d need to be able to predict how the mortality risks under the microbial mutiny hypothesis would differ from the null hypothesis.

Authors’ response: *Testing mortality risks may not be directly possible, because the hypothesis posits a secondary increase in virulence in situations where some background condition (aging, injury or infection) has generated a primary increase in virulence: the secondary effect therefore cannot be compared to healthy baseline mortality. We do not have sufficiently accurate prediction frameworks that could quantitatively estimate the primary increase in mortality (due to age or primary illness) to provide a comparison baseline for the detection or estimation of the secondary effect. We therefore believe that the testing of the hypothesis will have to rely on the discovery (and possible targeted manipulation) of the microbial mechanisms of “microbiome mutiny”, and now we discuss this more explicitly in the Testing the hypothesis section.*

Further, a better understanding of the nature of transmission and the population of ‘susceptibles’ could also be useful for understanding whether or not there is sufficient selection for such an adaptive response to evolve.

I think the authors’ paper would be more effective if they laid out some of the assumptions and ideas they introduce towards the end (e.g. in the Implications of the Hypothesis section)

Authors’ response: *We thank the reviewer for this excellent suggestion, and have now extended the list of assumptions/conditions that we believe are required to impose selection pressure for the evolution and maintenance of mechanisms of “microbiome mutiny”. We have added new text towards the end of the Presentation of the hypothesis section (last but one paragraph), and have summarized the list of criteria in a new *Table 1*, to highlight these conditions.*

and further clarified the difficulty in disentangling the causal effects of an increase in mortality rate of the host due to expected declines in immune system function or other aspect of senescence and a shift in symbiont strategies.

Authors’ response: *We have added further clarification to the Testing the hypothesis section.*

The reader would also benefit from some more information on how the authors think this increase in virulence would lead to an increase in transmission between hosts

Authors’ response: *Virulence and transmissibility can have a generic link through the rate of replication or titer of the pathogen in the host. If the pathogen grows to a higher titer, its concentration increases in the droplets or body fluids that mediate transmission, increasing the instantaneous rate of transmissibility. However, growing to higher titer requires more resources from the host (and all concentration-dependent symptoms also increase), which is likely to harm the host (i.e., increase virulence). We provide some specific examples (diarrhea, cough, bacteriuria), where increasing symptoms and/or pathogen titer can increase transmissibility.*

and why this wouldn’t be negated by the fact that transmission would likely be largely restricted to within a local social group which, as I understand, also generally share microbial symbionts. Otherwise, it’s hard to understand how this would be an ‘adaptive’ response on the part of the microbial community members.

Authors’ response: *We agree that the strategy of “microbiome mutiny” benefits the symbionts/pathogens only if they can be transmitted to host individuals that had not harbored them previously. We have added this criterion to the list of prerequisites for the evolution of microbiome mutiny, and have added citations to recent studies that assessed the current diversity of the microbiome in human populations.*

Major Criticisms

1. How might one disentangle the shift in the host’s allocation strategy (e.g. smaller allocation to immune response) and general aspects of senescence contributions to the increase in virulence vs. a change in symbiont’s behavior?
  Authors’ response: *We believe this can only be done by identifying the mechanisms of microbiome mutiny, and have added detailed discussions of this issue to the manuscript.*
2. I think it would be helpful to explain why you think there would be a positive relationship between virulence and transmission for the gut microbiome? What about the fact that in small clans, which likely represented the situation experienced historically by most human, the gut microbiome is strongly shared between members of the group? It seems like research looking at the effect of social network structure on the evolution of virulence is worth mentioning.
  Authors’ response: *We agree that these are important conditions for the validity of the hypothesis, and have modified the manuscript to acknowledge and evaluate these issues (see above and in the revised manuscript).*
3. Regarding whether the “Observations consistent” with your hypothesis listed at the bottom of page 4 causes of the increase in background mortality or responding to it; one way to differentiate b/w the two is that opportunistic pathogens would have a within-host net reproductive ratio (R0) that is less than one in a healthy host, but greater than 1 in aged hosts. A symbiont would have a within-host R0 > 1 in both healthy and aged hosts.
  Authors’ response: *We believe that the conditions for the conditional “microbiome mutiny” strategy are that (i) the symbiont (low-virulence) strategy should have an R0 greater than that of the pathogenic (high-virulence) strategy in a healthy host; (ii) the pathogenic strategy should have an R0 greater than that of the symbiont strategy in an aged or diseased host; and (iii) the overall R0 of this conditional strategy should be greater than one, given the density of healthy and aged/ill hosts in the population.*
4. The fact that there are many different symbionts and that there may be a positive feedback loop between them in terms of their shift from a beneficial or neutral relationship to a pathogenic relationship is a very important and interesting one.
  Authors’ response: *We thank the reviewer for his appreciation of the idea.*
5. The hypothesis put forth, the importance of ‘microbial mutiny’ seems like it would apply anytime the host has some increase in its mortality rate that is reflected by the physiological state within the host rather than just aging hosts. The authors mention this on page 5, but they may want to emphasize it more and do so earlier. Aging hosts would be an important category, but given how quickly the members of the microbiome can change with something as simple as a shift in diet, it seems like we might expect to see this behavior more often.
  Authors’ response: *We have added some new text to make sure that all possible causes of increasing background mortality (illness and injury, in addition to senescence) are mentioned at the appropriate points in the text.*
6. The authors seem to lump together 'pre-programmed’ responses of resident microbiome community members and evolutionary change of the community within the host. Do they think these categories are equivalent or just difficult to distinguish between? Do they have any reasons why they might expect one type of change over the other under a particular set of circumstances?
  Authors’ response: *This is an important distinction and we thank the reviewer for calling our attention to it. We have extended the paragraph describing the two alternatives (at the end of the Testing the hypothesis section) to show that the two mechanisms call for selection at different levels (between and within hosts) that might in some cases be connected.*
7. If the microbes are responding to their local environment, it is not clear to me why in vitro, rather than in vivo measures of virulence or gene expression would be that informative.
  Authors’ response: *In vivo virulence arises from the complex interplay of microbial and host factors, and, as discussed above, teasing out the microbial virulence components of increased mortality in individuals with compromised health status is likely to be very difficult (if at all possible).*
8. It would seem like modeling, not just experimental work, would help researcher understand the possible importance and interaction between other aspects related to aging besides an increase in mortality rate and changes in the microbial community.
  Authors’ response: *We agree that disentangling complex effects can benefit from modeling, although at the moment it is yet unclear to us how alternative mechanisms could be modeled and distinguished in the aging/disease associated increase of mortality.*
9. I am skeptical that antibiotics could be used to exclude a particular set of members of the microbiome given that recolonization appears to happen at a relatively high rate. A better understanding of the direct cues might instead be a more fruitful road to intervention.
  Authors’ response: *We agree that targeting the direct cues will eventually offer a better approach. Until then, however, antibiotics may still be useful during transient episodes of increased background mortality, as they need to control facultative pathogens only until the causes of the primary health condition are eliminated. Recolonization is not going to be a problem once the host condition stabilizes in the low-mortality selection regime that favors the low-virulence strategy of the symbiont.*
10. Theory would also be useful for understanding what conditions are conducive to a virulence positive feedback loop between microbial species.
  Authors’ response: *We fully agree that this aspect of the hypothesis is particularly amenable to modeling.*

Minor Criticisms

1. Consider defining lifetime reproductive fitness of the symbiont as its between host net reproductive ratio, the commonly used measure of pathogen fitness.
  Authors’ response: *We would like to keep our wording of “transmission over the entire life cycle of infection”: a slightly less precise but more intuitive formulation of the same concept, for the benefit of a broader audience.*
2. The authors state, “General theoretical considerations suggest that symbionts evolve to an optimal virulence. . . ” I would weaken this statement slightly. Theory indicates that selection will favor pathogens that have higher net reproductive Ratio. Whether it is maximize depends on factors such as effective population size, heterogeneity of environment over time and space, etc.
  Authors’ response: *We have replaced “to” with “towards” to acknowledge that symbionts will not always attain “optimal” virulence.*
  Similarly the authors state that “theory suggests that increasing mortality might increase the optimal virulence of a symbiont.” While that may be true, a more general statement could be mat that “increasing mortality might select for higher virulence of the host’s symbions”.
  Authors’ response: *We believe that the two statements are equivalent. “Optimal virulence” sets the direction of selection, but it does not guarantee the outcome of evolution (which is indeed influenced by other factors, as well).*
3. Is there any evidence that the pathogens causing UTIs are from the original host or transmitted from another host?
  Authors’ response: *The main causative organism is E. coli, and it is believed to be acquired typically from the gut microbiome of the original host. However, community-wide outbreaks of uropathogenic clonal groups have been reported* (see, e.g., [48]).
4. I like the term “microbiome mutiny” and would suggest introducing it earlier in the paper.
  Authors’ response: *We thank the reviewer for his kind comment and suggestion, and have included the expression in the title of the paper.*
5. Another sentence explaining ‘regime shifts’ in ecosystems would help readers unfamiliar with this idea.
  Authors’ response: *We have added an explanatory cause to introduce the concept.*
6. Regarding selection for counter-adaptations in the host. Life history theory would suggest that this selection would be strong for young hosts but decline with age, especially post-reproductive age. The authors mention reasons why this may not be the case in humans, but I would suggest the do so earlier.
  Authors’ response: *We agree with the importance of this issue, and mention the importance of post-reproductive survival in humans immediately after introducing the notion of possible counter-adaptations.*
7. References from the primary literature would also be preferable over Caspari’s article in Scientific American.
  Authors’ response: *We have replaced the reference.*
8. What is the source of “whosoever hath not, . . . ”?
  Authors’ response: *This excerpt is from the New Testament (Matthew 13:12). We have added the source in-line; however, it can also be moved into the formal list of References if that fits the format of the publication better.*
  *We are indeed thankful to the reviewer for the thorough constructive criticism of our manuscript. While not all issues could be included in the main text due to space constraints, we are very glad that the format of Biology Direct allows access for all readers to the important issues raised and discussed in the reviewer comments and our responses.*

## References

[1] Maynard Smith J, Szathmáry E (1995). The major transitions in evolution.

[2] Méthot P-O, Alizon S (2014). What is a pathogen? Towards a process view of host-parasite interactions. Virulence.

[3] Thomas F, Brown SP, Sukhdeo M, Renaud F (2002). Understanding parasite strategies: a state-dependent approach?. Trends Parasitol.

[4] Mideo N, Reece SE (2012). Plasticity in parasite phenotypes: evolutionary and ecological implications for disease. Future Microbiol.

[5] Alizon S, Hurford A, Mideo N, Van Baalen M (2009). Virulence evolution and the trade-off hypothesis: history, current state of affairs and the future. J Evol Biol.

[6] Anderson RM, May RM (1982). Coevolution of hosts and parasites. Parasitology.

[7] Ewald PW (1983). Host-parasite relations, vectors, and the evolution of disease severity. Annu Rev Ecol Syst.

[8] Ewald PW (1994). Evolution of infectious disease.

[9] Frank SA (1996). Models of parasite virulence. Q Rev Biol.

[10] Ponton F, Biron DG, Moore J, Moller AP, Thomas F (2006). Facultative virulence: a strategy to manipulate host behaviour?. Behav Processes.

[11] Human Microbiome Project Consortium (2012). Structure, function and diversity of the healthy human microbiome. Nature.

[12] Jones OR, Scheuerlein A, Salguero-Gomez R, Camarda CG, Schaible R, Casper BB (2014). Diversity of ageing across the tree of life. Nature.

[13] Sasaki A, Iwasa Y (1991). Optimal growth schedule of pathogens within a host: switching between lytic and latent cycles. Theor Popul Biol.

[14] Alizon S, van Baalen M (2005). Emergence of a convex trade-off between transmission and virulence. Am Nat.

[15] Axelrod R, Hamilton WD (1981). The evolution of cooperation. Science.

[16] Cerf-Bensussan N, Gaboriau-Routhiau V (2010). The immune system and the gut microbiota: friends or foes?. Nat Rev Immunol.

[17] Pilotto A, Franceschi M, Vitale D, Zaninelli A, Di Mario F, Seripa D (2008). The prevalence of diarrhea and its association with drug use in elderly outpatients: a multicenter study. Am J Gastroenterol.

[18] Stowe RP, Kozlova EV, Yetman DL, Walling DM, Goodwin JS, Glaser R (2007). Chronic herpesvirus reactivation occurs in aging. Exp Gerontol.

[19] Reese TA, Wakeman BS, Choi HS, Hufford MM, Huang SC, Zhang X (2014). Coinfection. helminth infection reactivates latent gamma-herpesvirus via cytokine competition at a viral promoter. Science.

[20] Gavazzi G, Krause KH (2002). Ageing and infection. Lancet Infect Dis.

[21] Muller LM, Gorter KJ, Hak E, Goudzwaard WL, Schellevis FG, Hoepelman AI (2005). Increased risk of common infections in patients with type 1 and type 2 diabetes mellitus. Clin Infect Dis.

[22] Scheffer M, Carpenter S, Foley JA, Folke C, Walker B (2001). Catastrophic shifts in ecosystems. Nature.

[23] Sohal RS, Orr WC (1992). Relationship between antioxidants, prooxidants, and the aging process. Ann N Y Acad Sci.

[24] Levine ME (2013). Modeling the rate of senescence: can estimated biological age predict mortality more accurately than chronological age?. J Gerontol A Biol Sci Med Sci.

[25] Cox CE (2012). Persistent systemic inflammation in chronic critical illness. Respir Care.

[26] Tower J (2009). Hsps and aging. Trends Endocrinol Metab.

[27] Zaborina O, Lepine F, Xiao G, Valuckaite V, Chen Y, Li T (2007). Dynorphin activates quorum sensing quinolone signaling in Pseudomonas aeruginosa. PLoS Pathog.

[28] Miller MB, Bassler BL (2001). Quorum sensing in bacteria. Annu Rev Microbiol.

[29] Rutherford ST, Bassler BL (2012). Bacterial quorum sensing: its role in virulence and possibilities for its control. Cold Spring Harb Perspect Med.

[30] Sekirov I, Russell SL, Antunes LC, Finlay BB (2010). Gut microbiota in health and disease. Physiol Rev.

[31] Leggett HC, Benmayor R, Hodgson DJ, Buckling A (2013). Experimental evolution of adaptive phenotypic plasticity in a parasite. Curr Biol.

[32] Song SJ, Lauber C, Costello EK, Lozupone CA, Humphrey G, Berg-Lyons D (2013). Cohabiting family members share microbiota with one another and with their dogs. Elife.

[33] Antunes LC, McDonald JA, Schroeter K, Carlucci C, Ferreira RB, Wang M (2014). Antivirulence activity of the human gut metabolome. MBio.

[34] Hawkes K, O’Connell JF, Blurton Jones N, Alvarez H, Charnov EL, Cronk L, Chagnon NA, Irons W (2000). The grandmother hypothesis and human evolution. Adaptation and human behavior: an anthropological perspective.

[35] Råberg L, Stjernman M, Demas GE, Nelson DJ (2012). The evolutionary ecology of infectious disease virulence. Ecoimmunology.

[36] Albuquerque P, Nicola AM, Nieves E, Paes HC, Williamson PR, Silva-Pereira I (2013). Quorum sensing-mediated, cell density-dependent regulation of growth and virulence in Cryptococcus neoformans. MBio.

[37] Zaborin A, Defazio JR, Kade M, Kaiser BL, Belogortseva N, Camp DG (2014). Phosphate-containing polyethylene glycol polymers prevent lethal sepsis by multidrug-resistant pathogens. Antimicrob Agents Chemother.

[38] Gay RT, Belisle S, Beck MA, Meydani SN (2006). An aged host promotes the evolution of avirulent coxsackievirus into a virulent strain. Proc Natl Acad Sci U S A.

[39] Margolis E, Levin BR (2007). Within-host evolution for the invasiveness of commensal bacteria: an experimental study of bacteremias resulting from Haemophilus influenzae nasal carriage. J Infect Dis.

[40] Classen DC, Evans RS, Pestotnik SL, Horn SD, Menlove RL, Burke JP (1992). The timing of prophylactic administration of antibiotics and the risk of surgical-wound infection. N Engl J Med.

[41] Angus DC, van der Poll T (2013). Severe sepsis and septic shock. N Engl J Med.

[42] Bindels LB, Delzenne NM (2013). Muscle wasting: the gut microbiota as a new therapeutic target?. Int J Biochem Cell Biol.

[43] Kaprio J, Koskenvuo M, Rita H (1987). Mortality after bereavement: a prospective study of 95,647 widowed persons. Am J Public Health.

[44] Cole SW (2014). Human social genomics. PLoS Genet.

[45] Akashi YJ, Nef HM, Mollmann H, Ueyama T (2010). Stress cardiomyopathy. Annu Rev Med.

[46] Lanter BB, Sauer K, Davies DG (2014). Bacteria present in carotid arterial plaques are found as biofilm deposits which may contribute to enhanced risk of plaque rupture. MBio.

[47] Periyakoil VS (2013). Frailty as a terminal illness. Am Fam Physician.

[48] Manges AR, Johnson JR, Foxman B, O’Bryan TT, Fullerton KE, Riley LW (2001). Widespread distribution of urinary tract infections caused by a multidrug-resistant Escherichia coli clonal group. N Engl J Med.

